# Supplementary material for: A Novel Immunofluorescence Assay for the Rapid Serological Detection of SARS-CoV-2 Infection
Source: Viruses. 2021 Apr 24;13(5):747. doi: 10.3390/v13050747 (PMC8146438; doi:10.3390/v13050747)
Supplement: Supplementary file 1 [file viruses-13-00747-s001.zip › viruses-1193136-SI.pdf]

SUPPLEMENTARY DATA

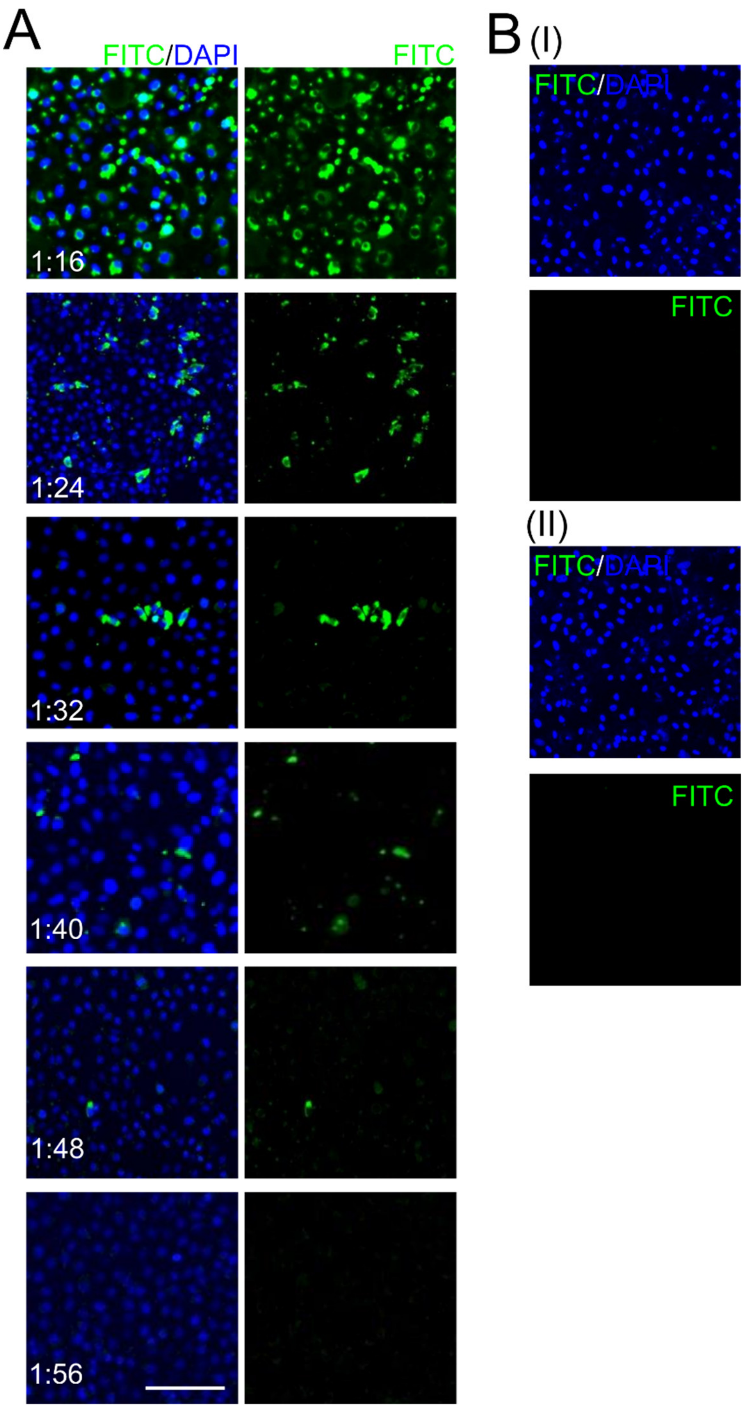

Figure S1: Immunofluorescence characterization of anti-SARS-CoV-2 IgG in SARS-CoV-2 spike stably expressing cells. Cells were imaged using an automated Evos FL Auto 2 microscopic system. (A) Indirect staining of A549 stable cells incubated with serum from a SARS-CoV-2 positive patient and FITC-conjugated (green) antibodies against human IgG. (B) (I) serum from a PCR confirmed negative donor sample and (II) spike-expressing stable A549 cells without serum. Nuclei were stained with DAPI (blue). Scale bar: 100 µm.

Figure S2. Nucleotide sequence of SARS-CoV-2.

>SARS-CoV-2_Jenner

TTCGTGTTCCTGGTGCTGCTGCCACTGGTGTCTAGCCAGTGTGTGAACCTGACCACCAGGACACAGCTGCCTCCAGCCTACACCAACAGC

TTCACCAGAGGCGTGTACTACCCAGACAAGGTGTTCAGATCCAGCGTGCTGCACTCTACCCAGGACCTGTTCCTGCCTTTCTTCAGCAAC

GTGACCTGGTTCCACGCCATCCACGTGTCCGGCACCAATGGCACCAAGAGATTCGACAACCCAGTGCTGCCCTTCAACGACGGAGTGTAC

TTTGCCAGCACCGAGAAGTCCAACATCATCAGAGGCTGGATCTTCGGCACCACACTGGACAGCAAGACCCAGAGCCTGCTGATCGTGAAC

AACGCCACCAACGTGGTCATCAAAGTGTGCGAGTTCCAGTTCTGCAACGACCCATTCCTGGGCGTCTACTACCACAAGAACAACAAGAGC

TGGATGGAAAGCGAGTTCCGGGTGTACAGCAGCGCCAACAACTGCACCTTCGAGTACGTGTCCCAGCCTTTCCTGATGGACCTGGAAGGC

AAGCAGGGCAACTTCAAGAACCTGCGCGAGTTCGTGTTCAAGAACATCGACGGCTACTTCAAGATCTACAGCAAGCACACACCTATCAAC

CTCGTGCGGGATCTGCCTCAGGGCTTCTCTGCTCTGGAACCACTGGTGGATCTGCCCATCGGCATCAACATCACCCGGTTTCAGACACTG

CTGGCCCTGCACAGAAGCTACCTGACACCTGGCGATAGCAGCTCTGGATGGACAGCTGGCGCCGCTGCCTACTATGTGGGATACCTGCAG

CCTCGGACCTTCCTGCTGAAGTACAACGAGAACGGCACCATCACCGACGCCGTGGATTGTGCTCTGGATCCTCTGAGCGAGACAAAGTGC

ACCCTGAAGTCCTTCACCGTGGAAAAGGGCATCTACCAGACCAGCAACTTCCGGGTGCAGCCCACCGAATCCATCGTGCGGTTCCCGAAT

ATCACCAATCTGTGCCCATTCGGCGAGGTGTTCAATGCCACCAGATTCGCCTCTGTGTACGCCTGGAACCGGAAGCGGATCAGCAATTGC

GTGGCCGACTACTCCGTGCTGTACAACTCCGCCAGCTTCAGCACCTTCAAGTGCTACGGCGTGTCACCTACCAAGCTGAACGACCTGTGC

TTCACAAACGTGTACGCCGACAGCTTCGTGATCCGTGGAGATGAAGTGCGGCAGATTGCACCTGGACAGACAGGCAAGATCGCCGACTAC

AACTACAAGCTGCCCGACGACTTCACCGGCTGTGTGATTGCCTGGAACAGCAACAACCTGGATAGCAAAGTCGGCGGCAACTACAATTAC

CTGTACCGGCTGTTCCGGAAGTCCAATCTGAAGCCCTTCGAGCGGGACATCTCCACCGAGATCTATCAGGCCGGCAGCACACCTTGTAAC

GGCGTGGAAGGCTTCAACTGCTACTTCCCACTGCAGTCCTACGGCTTTCAGCCCACAAATGGCGTGGGCTACCAGCCTTACAGAGTGGTG

GTGCTGAGCTTCGAGCTGCTGCATGCTCCTGCCACAGTGTGCGGCCCTAAGAAAAGCACCAATCTCGTGAAGAACAAATGCGTGAACTTC

AACTTCAACGGCCTGACCGGCACCGGCGTGCTGACAGAGAGCAACAAGAAGTTCCTGCCATTCCAGCAGTTCGGCCGGGATATCGCCGAT

ACCACAGATGCCGTCAGAGATCCTCAGACACTGGAAATCCTGGACATCACACCTTGCAGCTTCGGCGGAGTGTCTGTGATCACGCCTGGC

ACCAACACCAGCAATCAGGTGGCAGTGCTGTACCAGGACGTGAACTGTACCGAAGTGCCCGTGGCCATTCACGCCGATCAGCTGACACCT

ACATGGCGGGTGTACTCCACCGGCAGCAATGTGTTTCAGACCAGAGCCGGCTGTCTGATCGGAGCCGAGCACGTGAACAATAGCTACGAG

TGCGACATCCCTATCGGCGCTGGCATCTGCGCCTCTTACCAGACACAGACAAACAGCCCTAGACGGGCCAGATCTGTGGCCAGCCAGAGC

ATCATTGCCTACACAATGTCTCTGGGCGCCGAGAACAGCGTGGCCTACTCCAACAACTCTATCGCTATCCCGACCAACTTCACCATCAGC

GTGACCACAGAGATCCTGCCTGTGTCCATGACCAAGACCAGCGTGGACTGCACCATGTACATCTGCGGCGATTCCACCGAGTGCTCCAAC

CTGCTGCTGCAGTACGGCAGCTTCTGCACCCAGCTGAATAGAGCCCTGACAGGGATCGCCGTGGAACAGGACAAGAACACCCAAGAGGTG

TTCGCCCAAGTGAAGCAGATCTACAAGACGCCTCCTATCAAGGACTTCGGCGGCTTCAATTTCAGCCAGATTCTGCCCGATCCTAGCAAG

CCCAGCAAGCGGAGCTTCATCGAGGACCTGCTGTTCAACAAAGTGACACTGGCCGACGCCGGCTTCATCAAGCAGTATGGCGATTGTCTG

GGCGACATTGCCGCCAGGGATCTGATTTGCGCCCAGAAGTTTAACGGACTGACAGTGCTGCCTCCTCTGCTGACCGATGAGATGATCGCC

CAGTACACATCTGCCCTGCTGGCCGGCACAATCACAAGCGGCTGGACATTTGGAGCTGGCGCTGCCCTGCAGATCCCATTTGCTATGCAG

ATGGCCTACCGGTTCAACGGCATCGGAGTGACCCAGAATGTGCTGTACGAGAACCAGAAGCTGATCGCCAACCAGTTCAACAGCGCCATC

GGCAAGATCCAGGACAGCCTGAGCAGCACAGCAAGCGCCCTGGGAAAGCTGCAGGACGTGGTCAACCAGAATGCCCAGGCACTGAACACC

CTGGTCAAGCAGCTGTCTAGCAACTTCGGCGCCATCAGCTCTGTGCTGAACGATATCCTGAGCAGACTGGACAAGGTGGAAGCCGAGGTG

CAGATCGACAGACTGATCACCGGAAGGCTGCAGTCCCTGCAGACCTACGTTACCCAGCAGCTGATCAGAGCCGCCGAGATTAGAGCCTCT

GCCAATCTGGCCGCCACCAAGATGTCTGAGTGTGTGCTGGGCCAGAGCAAGAGAGTGGACTTTTGCGGCAAGGGCTACCACCTGATGAGC

TTCCCTCAGTCTGCGCCTCACGGCGTGGTGTTTCTGCACGTGACATACGTGCCCGCTCAAGAGAAGAATTTCACCACCGCTCCAGCCATC

TGCCACGACGGCAAAGCCCACTTTCCTAGAGAAGGCGTGTTCGTGTCCAACGGCACCCATTGGTTCGTGACCCAGCGGAACTTCTACGAG

CCTCAGATCATCACCACCGACAACACCTTCGTGTCTGGCAACTGCGACGTCGTGATCGGCATTGTGAACAATACCGTGTACGACCCTCTG

CAGCCCGAGCTGGACAGCTTCAAAGAGGAACTGGATAAGTACTTTAAGAACCACACAAGCCCTGACGTGGACCTGGGCGATATCAGCGGA

ATCAATGCCAGCGTCGTGAACATCCAGAAAGAGATCGACCGGCTGAACGAGGTGGCCAAGAATCTGAACGAGAGCCTGATCGACCTGCAA

GAACTGGGCAAGTACGAGCAGTACATCAAGTGGCCCTGGTACATCTGGCTGGGCTTTATCGCCGGACTGATTGCCATCGTGATGGTCACA

ATCATGCTGTGTTGCATGACCAGCTGCTGTAGCTGCCTGAAGGGCTGTTGTAGCTGTGGCTCCTGCTGCAAGTTCGACGAGGACGATTCT

GAGCCCGTGCTGAAGGGCGTGAAACTGCACTACACCTGA

>Spike_ Receptor _CO

GGATCCATGTTCCTGCTGACCACCAAGAGAACCATGTTCGTGTTCCTGGTGCTGCTGCCTCTGGTGAGCAGCCAGTGCGTGAATCTGACC

ACCAGAACCCAGCTGCCTCCTGCCTACACCAATAGCTTCACCAGAGGAGTTTATTATCCCGATAAGGTGTTCAGAAGTAGTGTATTACAT

AGTACCCAGGACCTGTTCCTACCTTTCTTCAGTAACGTGACCTGGTTCCACGCCATCCACGTGAGCGGCACCAATGGCACCAAGAGATTC

GACAATCCTGTGCTGCCTTTCAATGACGGCGTGTACTTCGCCAGCACCGAGAAGAGCAATATCATCAGAGGCTGGATCTTCGGCACCACC

TTGGATTCCAAGACTCAGAGCCTGCTGATTGTAAACAACGCTACAAATGTGGTGATCAAGGTGTGCGAGTTCCAGTTCTGCAATGACCCT

TTCCTGGGTGTTTATTATCATAAGAACAACAAGAGCTGGATGGAGAGCGAGTTCCGCGTATATTCGTCGGCTAATAATTGCACCTTCGAG

TACGTGAGCCAGCCTTTCCTGATGGACCTGGAGGGCAAGCAGGGCAATTTCAAGAATCTGAGAGAGTTCGTGTTCAAGAATATCGACGGC
TACTTCAAGATCTACAGCAAGCACACACCCATTAATCTGGTGAGAGACCTGCCTCAGGGCTTCAGCGCCCTGGAGCCTCTGGTGGACCTG
CCTATCGGCATCAATATCACCAGATTCCAGACCCTGCTGGCCCTGCACAGATCATATCTTACACCAGGCGATTCGTCAAGCGGTTGGACC
GCTGGAGCTGCGGCATATTACGTGGGCTACCTGCAGCCTAGAACCTTCCTGCTGAAGTACAATGAGAATGGTACGATAACCGACGCAGTT
GATTGTGCCCTGGACCCTCTGAGCGAGACCAAGTGCACCCTGAAGAGCTTCACCGTGGAGAAGGGCATCTACCAGACCAGCAATTTCAGA
GTGCAGCCTACCGAGAGCATCGTGAGATTCCCTAATATCACCAATCTGTGCCCTTTCGGCGAGGTGTTCAATGCCACCAGATTCGCCAGC
GTGTACGCATGGAACCGCAAGCGGATAAGCAATTGCGTGGCCGACTACAGCGTGCTGTACAATAGCGCCAGCTTCAGCACCTTCAAATGT
TATGGTGTTTCGCCAACAAAGCTGAATGACCTGTGCTTCACCAATGTGTACGCCGACAGCTTCGTGATCAGAGGCGACGAGGTGAGACAG
ATCGCGCCAGGGCAGACCGGCAAGATCGCCGACTACAATTACAAGCTGCCTGACGACTTCACCGGCTGCGTGATCGCGTGGAACTCTAAC
AATCTAGATTCGAAAGTTGGAGGCAATTACAATTACCTGTACAGACTGTTCAGAAAGAGCAATCTGAAGCCTTTCGAGAGAGACATCAGC
ACCGAGATCTACCAGGCCGGCAGCACACCGTGTAATGGCGTGGAGGGCTTCAATTGCTACTTCCCTCTGCAGAGCTACGGCTTCCAGCCT
ACCAATGGCGTGGGCTACCAGCCTTACAGAGTGGTGGTGCTGAGCTTCGAGCTGCTGCACGCTCCCGCTACCGTGTGCGGCCCTAAGAAG
AGCACCAATCTGGTGAAGAATAAGTGCGTGAATTTCAATTTCAATGGTCTAACTGGAACGGGCGTGCTGACCGAGAGCAATAAGAAGTTT
CTTCCCTTTCAACAATTCGGCAGAGACATCGCCGACACCACAGATGCTGTAAGAGACCCTCAGACCCTGGAGATCCTGGACATCACTCCG
TGTAGCTTCGGCGGCGTGAGCGTGATCACACCGGGTACCAATACCAGCAATCAGGTGGCCGTGCTGTACCAGGACGTGAATTGCACCGAG
GTGCCTGTGGCCATCCACGCCGACCAGCTGACTCCCACTTGGAGGGTATATTCCACGGGAAGCAATGTGTTCCAGACCAGAGCCGGCTGC
CTGATCGGCGCCGAGCACGTGAATAATAGCTACGAGTGCGACATCCCTATCGGCGCCGGCATCTGCGCCAGCTACCAGACCCAGACCAAT
AGCCCTAGAAGAGCCAGAAGCGTGGCCAGCCAGAGCATCATCGCCTACACCATGAGCCTGGGCGCCGAGAATAGCGTGGCCTACAGCAAT
AATAGCATCGCCATCCCTACCAATTTCACCATCAGCGTGACCACCGAAATATTACCAGTCTCCATGACCAAGACCAGCGTGGACTGCACC
ATGTACATCTGCGGCGACAGCACCGAGTGCAGCAATCTGCTGCTGCAGTACGGCAGCTTCTGCACCCAGCTGAATAGAGCCCTGACCGGC
ATCGCCGTGGAGCAGGACAAGAATACCCAGGAGGTGTTCGCCCAGGTGAAGCAGATCTACAAGACTCCGCCGATCAAGGACTTCGGCGGC
TTCAATTTCAGCCAAATACTCCCAGATCCAAGCAAGCCTAGCAAGAGGAGCTTCATCGAGGACCTGCTGTTCAATAAGGTGACCCTGGCC
GACGCCGGCTTCATCAAGCAGTACGGCGACTGCCTAGGTGATATTGCGGCAAGAGACCTGATCTGCGCCCAGAAGTTTAACGGTTTGACA
GTACTACCTCCTCTGCTGACCGACGAGATGATAGCACAATATACGTCGGCATTGCTCGCTGGCACGATCACATCGGGCTGGACTTTCGGC
GCCGGAGCAGCGTTGCAAATCCCTTTCGCCATGCAGATGGCCTACAGATTCAATGGCATCGGCGTGACCCAGAATGTGCTGTACGAGAAT
CAGAAGCTGATCGCCAATCAGTTCAATAGCGCCATCGGCAAGATCCAGGACAGCCTGAGCAGCACCGCCAGCGCCCTGGGCAAGCTGCAG
GACGTGGTGAATCAGAATGCCCAGGCCCTGAATACCCTGGTGAAGCAGCTGAGCAGCAATTTCGGCGCCATCAGTAGTGTACTCAACGAT
ATCCTGAGCAGACTGGACAAGGTGGAGGCCGAGGTGCAAATTGATCGTCTTATTACTGGCAGACTGCAGAGCCTGCAGACCTACGTGACC
CAGCAGCTGATCAGAGCCGCCGAGATCAGAGCCAGCGCCAATCTGGCCGCCACCAAGATGAGCGAGTGCGTGCTGGGCCAGAGCAAGAGA
GTGGACTTCTGCGGCAAGGGCTACCACCTGATGAGCTTCCCTCAGAGCGCTCCACATGGCGTGGTGTTCCTGCACGTGACCTACGTGCCT
GCCCAGGAGAAGAATTTCACCACCGCACCCGCAATCTGCCACGACGGCAAGGCCCACTTCCCTAGAGAGGGCGTGTTCGTGAGCAATGGC
ACCCACTGGTTCGTGACCCAGAGAAATTTCTACGAGCCTCAGATCATCACCACCGACAATACCTTCGTGAGCGGCAATTGCGACGTGGTG
ATCGGGATAGTCAATAATACTGTCTACGACCCTCTGCAGCCTGAGCTGGACAGCTTCAAGGAGGAGCTGGACAAGTACTTCAAGAATCAC
ACCAGCCCTGACGTGGACCTCGGTGATATTTCGGGAATCAATGCCAGCGTGGTGAATATCCAGAAGGAAATTGATCGGCTCAACGAAGTG
GCCAAGAATCTGAATGAGAGCCTGATCGACCTGCAGGAGCTGGGCAAGTACGAGCAGTACATCAAGTGGCCTTGGTACATCTGGCTGGGC
TTCATCGCCGGCCTGATCGCCATCGTGATGGTGACCATCATGCTGTGCTGCATGACCTCCTGTTGTTCCTGTTTGAAAGGGTGTTGTTCG
TGTGGGTCCTGCTGCAAGTTCGACGAGGACGACAGCGAGCCTGTGCTGAAGGGCGTGAAGCTGCACTACACCTGGAGCCACCCTCAGTTC
GAGAAGTGAGAATTC
